# Species richness, abundance, distributional pattern and trait composition of butterfly assemblage change along an altitudinal gradient in the Gulmarg region of Jammu & Kashmir, India

**DOI:** 10.1016/j.sjbs.2021.11.066

**Published:** 2021-12-03

**Authors:** Afaq Ahmad Dar, Khowaja Jamal, Muzamil Syed Shah, Mohd Ali, Samy Sayed, Ahmed Gaber, Hosny Kesba, Mohamed Salah

**Affiliations:** a Department of Zoology, Aligarh Muslim University, Aligarh 202002, Uttar Pradesh, India; b Department of Science and Technology, University College-Ranyah, Taif University, B.O. Box 11099, Taif 21944, Saudi Arabia; c Department of Biology, College of Science, Taif University, P.O. Box 11099, Taif 21944, Saudi Arabia; d Zoology and Agricultural Nematology Department, Faculty of Agriculture, Cairo University, Giza 12613, Egypt; e Department of Biology, Turabah University College, Taif University, B.O. Box 11099, Taif 21944, Saudi Arabia

**Keywords:** Biodiversity, Lepidoptera, Altitudinal gradients, Butterflies, Gulmarg

## Abstract

Despite enormous diversity, abundance, and role in ecosystem processes, little is known about how butterflies differ across altitudinal gradients. For this, butterfly communities were investigated along an altitudinal gradient of 2700–3200 m a.s.l, along the Gulmarg region of Jammu & Kashmir, India. We aimed to determine how the altitudinal gradient and environmental factors affect the butterfly diversity and abundance. Our findings indicate that species richness and diversity are mainly affected by the synergism between climate and vegetation. Alpha diversity indices showed that butterfly communities were more diverse at lower elevations and declined significantly with increase in elevation. Overall, butterfly abundance and diversity is stronger at lower elevations and gradually keep dropping towards higher elevations because floristic diversity decreased on which butterflies rely for survival and propagation. A total of 2023 individuals of butterflies were recorded belonging to 40 species, represented by 27 genera and 05 families. Six survey sites (S I- S VI) were assessed for butterfly diversity from 2018 to 2020 in the Gulmarg region of Jammu & Kashmir. Across the survey, Nymphalidae was the most dominant family represented by 16 genera and 23 species, while Papilionidae and Hesperiidae were least dominant represented by 01 genera and 01 species each. Among the six collection sites selected, Site I was most dominant, represented by 16 genera and 21 species, while Site VI was least dominant, represented by 04 genera and 04 species.

## 1 Introduction

Lepidoptera is the second largest order of Class Insecta. It comprises two sub-orders, i.e., Moths (Heterocera) and Butterflies (Rhaplocera), consisting of about 124 families (Kristensen et al., 2007). According to Van Nieukerken et al. (2011), butterflies count for 1.87% of the global insect fauna. Butterflies are great focal species because they are involved in various environmental processes (Arroyo et al., 1982). Due to their diverse and abundant nature, they are easily identified, collected and sampled round the year. Thus proving to the best models for population and ecology (Pollard, 1991). They are considered valuable indicators of forests due to widespread distribution, different land-use systems (Dar and Jamal, 2021-a., Schulze et al., 2004) and land cover types (Soga et al., 2015). Their pollination efficiency is higher than that of bees at higher elevations. They account for 75% of the floral visitors of the Asteraceae family, one of the most common plant families in Brazilian grasslands (Mota et al., 2016). Lepidopteran species are highly sensitive to climatic factors such as temperature, humidity, rainfall, and wind speed (Bhardwaj et al., 2012), affecting their distribution across environmental gradients seen in both mountainous and low-lying areas. They also have a close association with the vegetation, that they need for food and protection from predators, especially in the immature stages (Ferrer-Paris et al., 2013). The adults feed on floral nectar, due to this, they are less specialized to their host plant than the larvae. As a result, the distribution of butterfly species is linked to plant structure and community makeup (Carneiro et al., 2014). Availability of plant resources, habitat quality and quality of natural and semi-natural habitats are some of the factors that govern the distribution of butterflies. Mountains are ideal for exploring the effects of rising elevation and climate change on species diversity and abundance because they generate a variety of physical conditions in a relatively short distance. Patterns in species richness along altitudinal gradients are well-known ecological phenomena. However, there is a paucity of information about how ecological filtering processes affect the composition and features of butterfly assemblages at high altitudes. Altitudinal species gradients are shaped by long-term speciation processes, dispersal and extinction events (Mittelbach, 2010) and short-term ecological interactions with other organisms and the environment.

Many hypotheses provide a framework for understanding patterns of species richness and abundance along altitudinal gradients. The species energy theory predicts that as the altitude rises, species richness will decline due to increased energy availability and reduced population sizes at the mountain tops (Chown et al., 2012). Another hypothesis explains the decrease of biodiversity along altitude gradients with the species-area relationship (Lomolino, 2000). However, biodiversity does not always follow a linear pattern with increasing altitude and can peak at intermediate altitudes, possibly as a result of reduced biodiversity at low elevations due to human impact, highest productivity at mid-elevations, and the mid-domain effect, which explains a species richness peak at mid-elevation by stochastic effects of randomly distributed species that overlap more towards the centre of a geographical domain (Colwell et al., 2004). The presence or absence of any species is determined by the traits of the species. Species that live in similar environments and climates have similar traits. Cool-adapted species are moving northward and upward to avoid rising temperatures (Parmesan, 2006). Mountain-dwelling species moved their optimal elevation higher than non-mountain species (Lenoir et al., 2008). As a result, examining species traits that are sensitive to climatic and environmental variables, such as those found along altitudinal gradients, could help researchers better understand how species react to climate change (Diamond et al., 2011).

Although Gulmarg is very rich in biodiversity, only a few studies have been carried out to explore the butterfly fauna. Moore, (1874) reported 103 species of butterflies from the Gulmarg region of Jammu & Kashmir. Afterwards, Lang, (1868) explored the butterfly fauna of Gulmarg. Recently, Qureshi et al., (2013) investigated the butterfly fauna of Gulmarg and reported 31 species belonging to 8 families. Sharma & Sharma, (2021) conducted a study in Shiwaliks of the Jammu region and reported 118 species of butterflies represented by 81 genera and 6 families. Moreover, Sheikh et al., (2021) prepared a consolidated checklist of Rhaploceran fauna of Jammu & Kashmir, in which 308 species were reported. In this research paper diversity, abundance, distributional pattern and trait composition of butterflies in Gulmarg region of Jammu & Kashmir, were quantified over a 3-year period along an altitudinal gradient ranging from 2700 m a.s.l to 3200 m a.s.l. Results show that, richness and abundance is stronger at lower elevations and keeps on decreasing as elevation increases. Traits of butterflies were also studied, it showed that most of the traits of butterflies at low elevation kept on changing as elevation increases for e.g., large wing sized butterflies were dominant at higher elevation and small wing sized butterfly species were dominant at lower elevation. Overall, diversity and abundance is not only affected by increasing elevation, but environmental factors in synergism with vegetation change the community structure of butterflies.

## 2 Materials & methods

### 2.1 Study area and survey sites

**Fig. 1:**
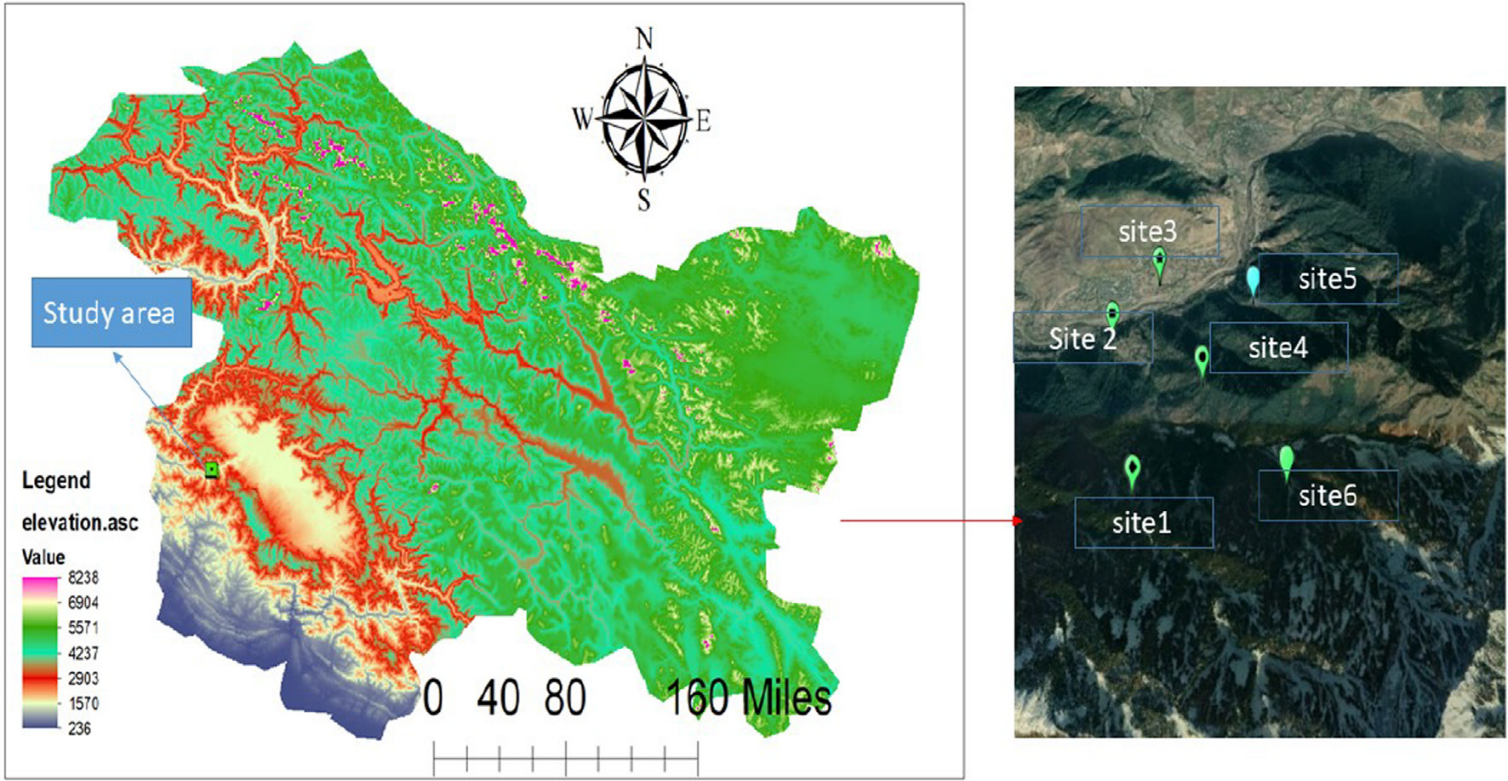
Sampling area and survey site of Gulmarg, Jammu and Kashmir.

Gulmarg is a famous hill station in the Baramulla district of Jammu & Kashmir, located at 2650 m a.s.l in the Pir Panjal Range of the western Himalayas. In Gulmarg, Six Survey sites, SI- S VI, were selected for accessing butterfly diversity (As shown in Fig. 1). These sites were selected according to the elevation. S I was located by 2700 m a.s.l, while as Site VI was located at 3200 m a.s.l. Glacial deposits, lacustrine deposits, and moraines cover shales, sandstones, schists, and other types of rocks found in it. During the spring and summer, the natural meadows of gulmarg are covered in snow during the winter. Enclosed parks and small lakes are interspersed throughout the fields, which are flanked by verdant pine and forests.

### 2.2 Data collection

The field trips of the study area were undertaken from March 2018 to November 2020 to explore the butterfly diversity. In study area, six survey sites, (SI- S VI) were accessed for butterfly diversity and abundance. In each survey site, GPS coordinates and above sea level length was measured by GPS Garmin Etrex. The butterflies were collected by sweeping net, followed by the photography of them. None of the butterfly species were killed during the collection.

### 2.3 Identification

The identification of butterflies was carried out with the help of identification keys, standard reference keys, available literature viz., (Smetacek, 2018, Kehimkar, 2016, Wynter-Blyth, 1957, Evans, 1932).

### 2.4 Diversity indices

Shannon diversity index

The Shannon index is a metric for determining the diversity and abundance of species. It's calculated using the following formula:

$$H=-\sum_{i=1}^{s}p_{i}*\left(\ln p_{i}\right)$$

Where:

H = Shannon index for species diversity,

S = Number of species,

P_i_ = Proportion of total sample belonging to the i^th^ species, and

ln = Natural log

Simpson’s diversity Index

$$\mathrm{SI}=\sum \;pi^{*}pi$$

Where:

SI = Simpson’s Index of species diversity,

S = No. of species, and

pi = proportion of total sample belonging to the i^th^ species

Brillouin index

The Index is more sensitive to species abundance

It’s calculated as

$$HB=\frac{ln(N!-\sum ln(n!)}{N}$$

Where HB is Brillouin index

N is the total number of individuals in the sample

n is the number of individual of species i

ln(x) refers to natural logarithm of x

Fisher’s alpha index

It's a tool for determining the diversity of a population.

It’s calculated by the following formula

$$S=a*ln(1+\frac{n}{a}$$

Where S is equal to number of taxa

N is number of individuals

And a is fisher’s alpha

## 3 Results

**Fig. 2:**
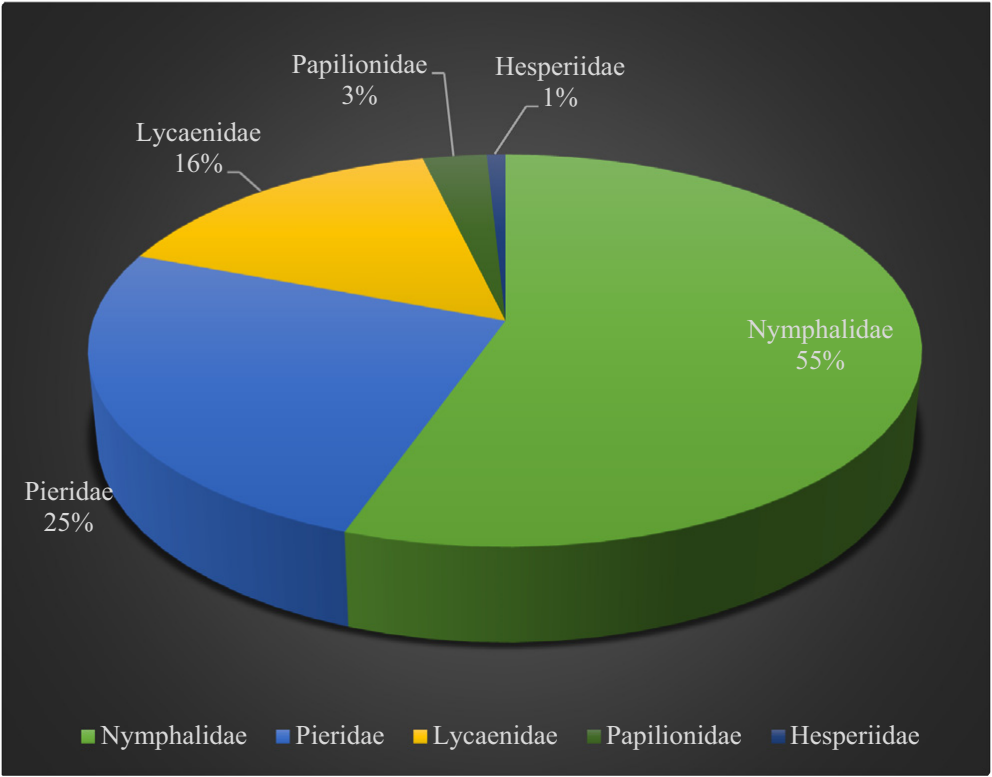
The species abundance of butterfly fauna in relation to their families in Gulmarg region of Jammu & Kashmir.

**Fig. 3:**
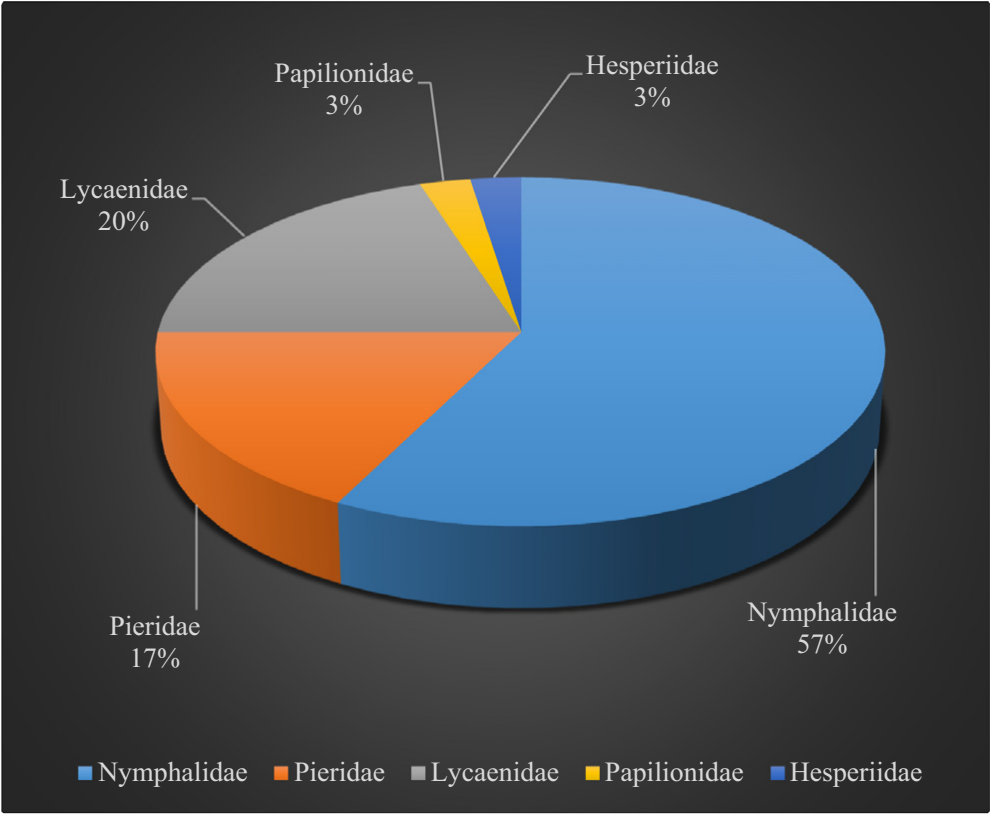
The species richness of butterfly fauna in relation to their families in Gulmarg region of Jammu & Kashmir.

Altogether we recorded 2023 individuals of butterflies, belonging to 40 species under 27 genera and 5 families. The different species of butterflies are presented in Table 1. Out of 2023 individuals of butterflies, 1121 individuals belong to the family Nymphalidae, followed by 513 individuals belonging to Pieridae, 314 individuals belong to Lycaenidae, 58 individuals belong to Papilionidae, and 17 individuals belong to Hesperiidae (As shown in Fig. 2). The Nymphalidae family had the most species, with 23, followed by Lycaenidae with 8, Pieridae with 7, Papilionidae and Hesperiidae with 1 each (As shown in Fig. 3). The Nymphalidae family had the most genera 16, followed by Lycaenidae with 5 genera, Pieridae with 04 genera, Papilionidae and Hesperiidae with 01 genera each.

**Table 1:** Butterfly fauna recorded from the survey sites of Gulmarg region of J&K.

S. No	Species	Total no. of individuals	Family	Author & Year
01	*Junonia iphita siccata*	55	Nymphalidae	(Fruhstorfer, 1900)
02	*Junonia orithya swinhoei*	48	Nymphalidae	(Butler, 1885)
03	*Junonia hiertha*	37	Nymphalidae	(Fabricius, 1798)
04	*Aulocera brahminus*	65	Nymphalidae	(Blanchard, 1853)
05	*Aulocera padma*	76	Nymphalidae	(Kollar, 1844)
06	*Aulocera swaha garuna*	46	Nymphalidae	(Frushstorfer, 1911)
07	*Hyponephele coenonympha*	66	Nymphalidae	(C. & R. Felder, 1867)
08	*Hyponephele kashmirica*	75	Nymphalidae	(Moore, 1892)
09	*Vanessa cardui*	68	Nymphalidae	(Linnaeus, 1758)
10	*Vanessa indica*	89	Nymphalidae	(Herbst, 1794)
11	*Argynnis hyperbius*	38	Nymphalidae	(Linnaeus, 1763)
12	*Argynius childreni*	37	Nymphalidae	(Kollar, 1848)
13	*Aglais caschmirensis*	30	Nymphalidae	(Kollar, 1884)
14	*Melanitis phedima galkissa*	31	Nymphalidae	(Fruhstorfer, 1911)
15	*Libythea lepita*	56	Nymphalidae	(Moore, 1858)
16	*Kaniska canace*	29	Nymphalidae	(Linnaeus, 1763)
17	*Neptis hylas kamarupa*	33	Nymphalidae	(Moore, 1875)
18	*Callerebia nirmala daksha*	90	Nymphalidae	(Moore, 1874)
19	*Kirinia eversmanni cashmirensis*	37	Nymphalidae	(Moore, 1874)
20	*Lasiommata scharka*	20	Nymphalidae	(Kollar, 1844)
21	*Nymphalis vaualbum*	15	Nymphalidae	(Denis & Schiffermuller, 1775)
22	*Hypolimnas misippus*	31	Nymphalidae	(Linnaeus, 1764)
23	*Issoria lathonia issaea*	49	Nymphalidae	(Gray, 1864)
24	*Colias fieldi*	74	Pieridae	(Menetries, 1855)
25	*Colias erate*	66	Pieridae	(Esper, 1805)
26	*Pieris brassicae nepalensi*	102	Pieridae	(Gray, 1846)
27	*Pieris canidia indica*	94	Pieridae	(Evans, 1926)
28	*Aporia nabellica*	46	Pieridae	(Bosiduval, 1836)
29	*Aporia soracta*	38	Pieridae	(Moore, 1857)
30	*Pontia daplidice moorei*	93	Pieridae	(Rober, 1907)
31	*Rapala nissa*	35	Lycaenidae	(Moore, 1857)
32	*Rapala selira*	47	Lycaenidae	(Moore, 1847)
33	*Celastrina gigas*	24	Lycaenidae	(Hemming, 1928)
34	*Celastrina argiolus kollari*	33	Lycaenidae	(Westwood, 1852)
35	*Lycaena phlaeas baralacha*	48	Lycaenidae	(Linnaeus, 1761)
36	*Lycaena kasyapa*	34	Lycaenidae	(Moore, 1865)
37	*Aricia agestis nazira*	17	Lycaenidae	(Moore, 1865)
38	*Lampides boeticus*	76	Lycaenidae	(Linnaeus, 1767)
39	*Papilio machaon asiatica*	58	Papilionidae	(Menetries, 1855)
40	*Pelopidas mathias*	17	Hesperiidae	(Fabricius, 1798)
Total		2023

### 3.1 Diversity indices along altitudinal gradient

When diversity indices are applied, interpreting species distribution becomes easier. According to Barrantes and Sandoval, (2009), using multiple indices helps eliminate drawbacks of the individual index. Shannon’s Index describes species diversity. Maximum species diversity was observed for Site I (2.92), and minimum diversity for Site VI (1.261). When Shannon’s index is calculated, a weighted geometric mean of the proportional abundances is employed. Thus, it reflects the logarithm of actual diversity observed and is used frequently. Simpson’s index uses the weighted arithmetic mean or proportional abundances and describes species richness and evenness. Thus, a high Simpson’s index suggests higher species richness and evenness. The maximum value for Simpson’s index was calculated for Site I (0.9413) and the minimum for Site VI (0.6784). This suggests that the species richness at Site I is definitely high and there is an evenness to the species distribution too.

The following index calculated was Brillouin’s index. According to Magurran, (1988), this diversity index serves better when there is no surety for the randomness of the sample. Thus, to eliminate any biases raised unknowingly, we employed this index. The maximum value for this index was calculated again for Site I (2.859) and the minimum for Site VI (1.195). Lastly, Fisher’s alpha was the fourth alpha diversity index employed. In cases where sample sizes vary a lot, Fisher’s alpha has a good discriminating capability. As the sample size for all the sampling stations varied, this index was used. Maximum value for this index was calculated for Site I (3.881) and the minimum for Site VI (0.8299). Thus, all the four alpha diversity indices confirm that maximum alpha diversity was observed at Site I and minimum at Site VI (As shown in Table 2). A Similar type of research was carried out by (Ahmad Dar et al., 2021, Dar and Jamal, 2021-b., Naz et al., 2021, Liu et al., 2019, Bokhorst et al., 2018, Qing et al., 2015, Illig et al., 2010, Cutz-Pool et al., 2010). They also concluded that, diversity indices show a decrease in diversity and abundance decreases along an altitudinal gradient.

**Table 2:** Diversity indices for survey sites.

Diversity Indices	Site I	Site II	Site III	Site IV	Site V	Site VI
No. of Taxa	21	16	12	08	06	04
Individuals sites	865	403	366	148	139	102
Simpson’s Index	0.9413	0.9277	0.8556	0.823	0.798	0.6784
Shannon-Weiner Index	2.92	2.692	2.138	1.878	1.679	1.261
Brillouin’s Index	2.859	2.603	2.069	1.778	1.599	1.195
Fisher’s alpha	3.881	3.331	2.38	1.812	1.277	0.8299

### 3.2 Change in abundance of butterfly community along altitudinal gradient

In terms of abundance, Site I, Site II, Site III, Site IV, Site V and Site VI were represented by 865, 403, 366, 148, 139 and 102 individuals (As shown in Table 3). In site I, *Hyponephele kashmirica* was the most dominant (75) and *Kirinia eversmanni cashmirensis* was the least abundant (14). In Site II, *Pontia daplidice moorei* was the most dominant (33), while as *Melanitis phedima galkissa* and *Kaniska canace* were least dominant (11 each). In Site III, *Vanessa indica* was the most abundant represented by 81 individuals, while as *Nymphalis vaualbum* was the least dominant represented by 06 individuals. In Site IV, *Aulocera padma* was the most dominat represented by 34 individuals while as *Celastrina argiolus kollari* was the least dominant represented by 06 individuals. In Site V, *Argynius children* was the most dominant represented by 37 individuals, while as *Hypolimna misippus* was the least dominant represented by 10 specimens. In Site VI, *Isooria lathonia issaea* was the most dominant with 49 individuals, while as *Aulocera swaha garuna* was the least dominant with 16 individuals. Across the survey, *Pieris brassicae nepalensi* was the most dominant with 102 individuals, while as *Nymphalis vaualbum* was the least dominant with 15 individuals.

**Table 3:** Butterfly fauna at survey sites (S I- S VI) showing elevation, abundance and GPS Coordinates.

Sites	Elevationm a.s.l	Species found	Total no. of Individuals	GPS Coordinates
I	2700	1. *Colias fieldi* 2. *Colias erate* 3. *Pieris brassicae nepalensi* 4. *Pieris canidia indica* 5. *Aporia nabellica* 6. *Aporia soracta* 7. *Junonia iphita siccata* 8. *Junonia orithya swinhoei* 9. *Hyponephele coenonympha* 10. *Hyponephele kashmirica* 11. *Argynnis hyperbius* 12. *Melanitis phedima galkissa* 13. *Libythea lepita* 14. *Kaniska canace* 15. *Nymphalis vaualbum* 16. *Lampides boeticus* 17. *Celastrina gigas* 18. *Pontia dalpidice moorie* 19. *Pelopidas mathias* 20. *Callerebia nirmala daksha* 21. *Kirinia eversmanni cashmirensis*	50 45 60 48 46 38 32 35 66 75 23 20 56 18 9 76 24 60 17 53 14	34°11′20.58′′N 74°12′3.22′′E
II	2800	1. *Colias fieldi* 2. *Colias erate* 3. *Pieris brassicae nepalensi* 4. *Pieris canidia indica* 5. *Rapala selira* 6. *Rapala nissa* 7. *Lycaena phlaeas baralacha* 8. *Lycaena kasyapa* 9. *Jononia ipitha siccata* 10. *Junonia orithya swinhoei* 11. *Celastrina argiolus kollari* 12. *Pontia daplidice moorei* 13. *Argynnis hyperbius* 14. *Melanitis phedima galkissa* 15. *Kaniska canace* 16. *Callerebia nirmala daksha*	24 21 30 32 47 25 30 34 23 13 17 33 15 11 11 37	34°11′12.96′′N 74°10′56.56′′E
III	2900	1. *Pieris brassicae nepalensi* 2. *Pieris canidia indica* 3. *Aulocera brahminus* 4. *Aulocera padma* 5. *Vanessa cardui* 6. *Vanessa indica* 7. *Kirinia eversmanni cashmirensis* 8. *Nymphalis vaulbum* 9. *Lycaena phlaeas baralacha* 10. *Rapala nissa* 11. *Celastrina argiolus kollari* 12. *Junonia hiertha*	12 14 65 42 68 81 10 6 11 10 10 37	34°12′1.08′′N 74°11′27.92′′E
IV	3000	1. *Lycaena phlaeas baralacha* 2. *Kirinia eversmanni cashmirensis* 3. *Aulocera padma* 4. *Argynnis hyperbius* 5. *Celastrina argiolus kollari* 6. *Hypolimnas misippus* 7. *Papilao machaon asiatica* 8. *Aglais cascmirensis*	7 13 34 8 6 21 40 19	34°12′54.07′′N 74°10′10.27′′E
V	3100	1. *Argynius childreni* 2. *Neptis hylas kamarupa* 3. *Aglais cascmirensis* 4. *Hypolimnas misippus* 5. *Papilio machaon asiatica* 6. *Aulocera swaha garuna*	37 33 11 10 18 30	34°13′12.65′′N 74°10′6.28′′E
VI	3200	1. *Aricia agestis nazira* 2. *Issoria lathonia issea* 3. *Lasiommata scharka* 4. *Aulocera swaha garuna*	17 49 20 16	34°13′39.49′′N 74°9′28.67′′E

In line with the results that we observed, that total butterfly abundance shows significant decline with an increase in altitude, which, as per the results of some earlier studies carried out by (Afzal et al., 2021, Sharma and Sharma, 2021, Antão et al., 2020, Leingärtner et al., 2014, Diamond et al., 2011).

### 3.3 Change in species richness of butterfly community along elevation gradient

**Fig. 4:**
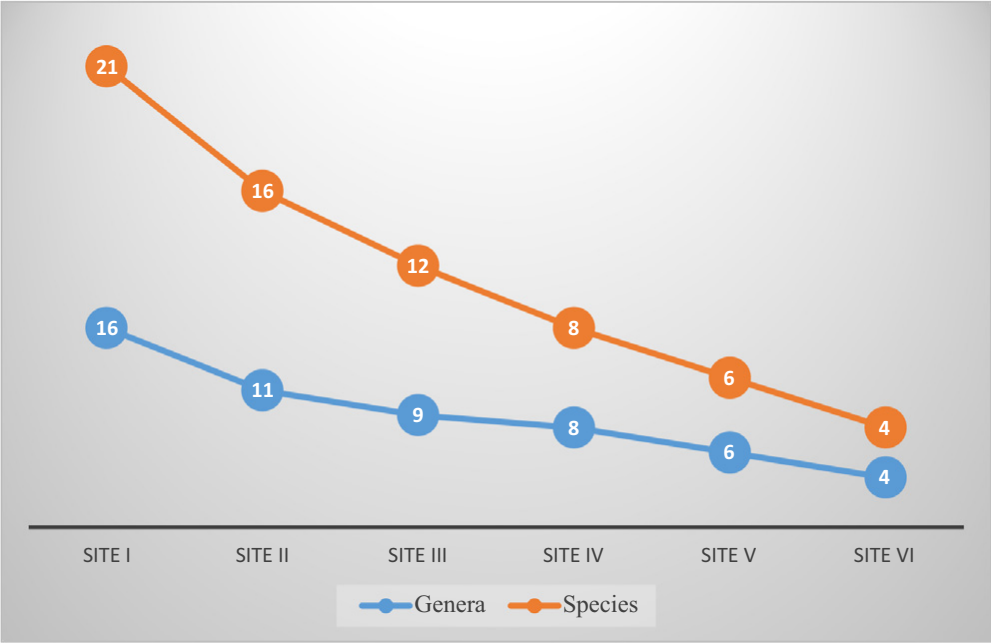
Number of genera and species in relation of survey sites (SI- SVI).

Among the survey sites, Site I with an elevation of 2700 m a.s.l was most dominant with 16 genera and 21 species, followed by Site II with an elevation of 2800 m a.s.l represented by11 genera and 16 species, Site III with an elevation of 2900 m a.s.l represented by 09 genera and 12 species, Site IV with an elevation of 3000 m a.s.l represented by 08 genera and 08 species, Site V with elevation of 3100 m a.s.l represented by 06 genera and 06 species, while as Site VI was least dominant with an elevation of 3200 m a.s.l represented by 04 genera and 04 species (As shown in Table 3 and Fig. 4). In Site I, 11 species belong to family Nymphalidae, 7 species to Pieridae, 2 species to Lycaenidae and 1 species to Hesperiidae. In Site II, 6 species belong to Nymphalidae, 5 species to Pieridae and 5 species to Lycaenidae. In Site III, 6 species belong to Nymphalidae, 2 species to Pieridae and 2 species to Lycaenidae. In Site IV, 5 species belong to Nymphalidae, 2 species to Lycaenidae and 1 species to Papilionidae. In Site V, 5 species belong to Nymphalidae and 1 species to Papilionidae. Lastly, in Site VI, 3 species belong to Nymphalidae and 1 species to Lycaenidae.

Our results are in agreement with the research carried out by (Sharma and Sharma, 2021, Singh and Sondhi, 2016, Sondhi and Kunte., 2016, Chown et al., 2012, Joshi et al., 2008, Joshi and Arya, 2007, Ockinger et al., 2006). They also concluded that that total butterfly species richness significant decline with an increase in altitude.

### 3.4 Impact of environmental gradients on species richness and abundance along an elevation gradient

Biodiversity gradients are the outcome of several temporal and spatial dimensions of evolutionary and ecological processes (Ricklefs and Jenkins, 2011). Abiotic variables, such as climate and topographic gradients, are connected with the majority of these biological processes (Stein et al., 2014). The climate and topography gradients are inextricably linked. Climate change can have an indirect impact on species diversity due to its impacts on vegetation (Stein et al., 2014). While topography has an impact on both climate and vegetation, it also has an impact on species diversity. (Stein et al., 2014, Ruggiero and Hawkins, 2008). We employed four variables to account for climatic factors: (i) average temperature, (ii) average rainfall, (iii) average relative humidity, and (iv) average rainy days are represented from 2018 to 20. All climatic variables were downloaded from Indian Meteorological Department, Govt. of India (“IMD | Home,” n.d.).

The average temperature was observed maximum in the month of August and minimum in November. As the monsoon ends in the month of August, this suggests that maximum species richness and abundance are related to the temperature. Another variable is rainfall; the maximum rainfall is observed in the month of monsoon, which falls in the month of July and August. This maximum rainfall is indirectly associated with the growth of floral diversity, on which butterflies and other taxa feed, survive and propagate. The final variable is average relative humidity. It's a well-known fact that the more the relative humidity, the more will be richness and abundance. The maximum value of average relative humidity is seen in July and August, while as minimum average relative humidity is seen in November. Our findings were also in agreement with (Lee et al., 2014, Montero-Munoz et al., 2013, Valtonen et al., 2013, Camero et al., 2007, Clark et al., 2007). They concluded that environmental gradients directly or indirectly influence the butterfly richness and abundance.

### 3.5 Trait composition along an elevational gradient

In this step, we analyzed whether the trait composition of butterflies such as wing-length, abdomen size, body coloration and dispersal of butterfly assemblages change with increasing altitude or not? We observed large winged butterflies were found at higher altitudes than lower altitudes. Along the increasing altitudinal gradient from Site I to Site VI, the percentage of small wing size butterflies shows an increase but started declining after site III. In contrary to medium wing size butterflies, showed a considerable decrease in wing size on altitudinal increase. The percentage of large wing size butterflies increase with increasing elevation. Therefore, we can conclude that wing size decreases as elevation increases. Similarly, abdomen size increases on moving from low elevation to high elevation. At high altitude, most of the catches found were having large abdomen sizes. Lastly, on observing the coloration of the butterflies, we found that altitudinal gradients have no effect on coloration. At lower altitudes, some butterflies were brightly coloured and had a wide variety of coloration, and at high altitude, some were brightly colored and rest were dull coloured. Our findings are in agreement with (WAGNER et al., 2011, Gaston et al., 2008); Karl et al., 2008, Berner et al., 2004).

## 4 Discussion

Biodiversity is changing dramatically and quickly all over the world. The significance of this transition, however, is unclear, as community-level alterations may obscure differences in individual population trajectories. For decades, ecologists and biogeographers have been fascinated by the richness, abundance, and distribution of species along elevational gradients (Rohde, 1999). In a range of habitats and taxa, multiple studies have observed species distribution along elevational and latitudinal gradients, and many mechanisms have been proposed to explain spatial variation in species richness (Szewczyk and McCain, 2016). The factors that underpin species distribution over elevational gradients, on the other hand, remain poorly understood. It is widely accepted that species diversity decreases as elevation rises, such declines are rarely clear. Several quantitative studies of diverse taxa have discovered evidence for four main species diversity patterns along elevational gradients: monotonic decrease, low plateau, low plateau with a mid-elevational peak, and unimodal mid-elevational peak, with the last being the most common (Grytnes and McCain, 2007). During the present investigation, Family Nymphalidae was found to dominate the fauna, represented by 1121 individuals, under 16 genera and 23 species, followed by Pieridae represented by 315 individuals under 4 genera and 7 species, Pieridae with 314 individuals under 5 genera and 8 species, Papilionidae with 58 individuals under 01 genera and 01 species, and Hesperiidae with 17 individuals under 01 genera and 01 species. Across the survey, 2023 individuals were collected belonging to 40 species under 27 genera and 05 families.

Environmental variables such as climate and vegetation properties have been used to explain animal species richness and abundance distribution patterns (Mittelbach, 2010). Environmental gradients (EG) such as temperature, humidity, rainfall and vegetation cover are the potential drivers of broad-scale patterns of species richness and abundance. From 2700 to 3200 m a.s.l, we looked into the influence of environmental gradients and vegetation on butterfly distribution. We assessed the strength of the association among butterflies, vegetation, and climate. Among the environmental conditions that shift strongly with elevation is temperature, which gradually decreased with elevation and proved detrimental for the butterfly community. Butterflies survival and reproduction are highly dependent on temperature variations. Any deviation from the optimal temperature causes substantial inhibition and propagation. As evident, temperature decreases as altitude increases, which decreases the species richness and abundance. Similar results are also shown by rainfall. As the elevation increases, humidity decreases, which hinders the growth of vegetation, on which butterflies rely for survival and propagation. Therefore, species richness and abundance decrease.

Furthermore, altitudinal climatic circumstances substantially constrain the availability and turnover of basal resources, vegetation cover is discovered to have a considerable impact on the butterfly community and can be seen as nature's own field trials. While studying the environmental gradients, a linear decrease of species richness and abundance was observed along the altitudinal gradient. This linear decrease may be due to the non-favorable environmental conditions, which doesn’t support the propagation of butterfly species. Results depict that, Site I was having the highest species richness (21 species), followed by Site II (16 species), Site III (12 species), Site IV (8 species), Site V (6 species) and Site VI (4 species). This linear decrease of butterfly species is due to the direct influence of environmental gradients, which doesn’t support species richness at higher altitudes. As already described, Shannon’s diversity index reflects diversity. Among the sites, Site I was showing maximum species diversity and Site VI was least diversity. Similarly, Simpson’s Index, Brillouin’s index and Fisher’s alpha showed maximum values in Site I and the least value in Site VI.

Studies involving the conservation of butterfly diversity are vital as Lepidoptera are an integral part of the food chain connecting autotrophs and heterotrophs. They are also well-known ecological indicators, sensitive to climatic and ecological changes and respond quickly to the stratification of the vegetation in terms of temperature, wind, light and humidity (Dar and Jamal, 2021). Moreover, studying the diversity of butterflies provide us with insight information about changes in abundance, evenness and richness affected by vegetation and species interactions and landscapes. The information about the species diversity, richness and abundance help in constructing conservation measures and management practices.

## 5 Conclusion

Our findings showed that in the Gulmarg region of Jammu & Kashmir, butterflies species richness and abundance are decreasing linearly as the elevation increases. This linear decrease is the resultant of decrease in vegetation assemblages along with other environmental factors which act along the altitudinal gradient. Thus we can conclude that, butterfly species are abundant at the lower elevation and keeps on decreasing as the elevation decreases. Alpha diversity indices are also in agreement with our results. Moreover, this linear decrease is not only due to the elevation, but environmental factors such as temperature, rainfall, humidity are also acting along with vegetation.

## Declaration of Competing Interest

The authors declare that they have no known competing financial interests or personal relationships that could have appeared to influence the work reported in this paper.
